# Aged care employment and the productivity commission: Fixing the data gaps may be the most useful thing it can do

**DOI:** 10.1111/ajag.13113

**Published:** 2022-07-08

**Authors:** Diane Gibson

**Affiliations:** ^1^ Health and Ageing, Faculty of Health University of Canberra Bruce Australian Capital Territory Australia

**Keywords:** employment insecurity, health services for the aged, health workforce, nursing homes, precarious employment

## Abstract

**Objective:**

To explore the gaps and anomalies in Australia's national aged care workforce data with a particular focus on casualisation and insecure employment in residential aged care.

**Methods:**

Secondary analysis of data from the National Aged Care Workforce Census and Surveys, the Aged Care Workforce Census and the Australian Bureau of Statistics Characteristics of Employment Survey.

**Results:**

There are significant and disturbing gaps in our knowledge of the aged care workforce deriving from disruptions to the time series as a result of methodological changes, reduced reliability resulting from declining response rates and the historical weighting system. Scope is also a critical factor due to data inadequacies relating to a non‐Pay As You Go (non‐PAYG) workforce and regarding the use of minimum hours contracts. This reduces our understanding of insecure employment.

**Conclusions:**

Australia needs better quality and more reliable data on its aged care workforce if the labour shortages confronting the sector are to be better understood and addressed. There is a critical need to determine the optimum strategy to obtain such data, whether by specific research projects of sufficient scale to accurately document the scale and scope of these issues, or in creative strategies to make use of automatically generated data.

**Policy Impact**

The inadequacies and anomalies in our national aged care workforce data have been neglected for too long. This issue needs to be recognised and addressed, and the extent of insecure employment documented if the growing crisis in staff attraction and retention is to be ameliorated.

## 1 INTRODUCTION

Australia's aged care workforce was the subject of a great deal of attention throughout the Royal Commission into Aged Care Quality and Safety. By 2021 when the Commission's main recommendations were released,^1^ COVID‐19 had brought home the inadequacies of staffing in residential aged care, with a plethora of stories of resident deaths, breaches of human rights and failures to deliver basic care.^2^ While the Royal Commission may have spiked public awareness, the problems with the aged care workforce have been raised in numerous national reviews and inquiries since 2005,^3^ and between 2018 and 2020 five official reports have put forward workforce‐related recommendations.^4^, ^5^, ^6^, ^7^, ^8^

The Commission's recommendations spanned educational qualifications and skills development (Recommendations 79–83), minimum qualifications and regulation of the personal care workforce (Recommendations 77, 78), minimum staffing standards in residential care (Recommendation 86), remuneration (Recommendations 84, 85), employment status and related labour force standards specifically relating to direct employment (Recommendation 87) and workforce planning, including obtaining up‐to‐date data on the aged care workforce (Recommendation 75).^1^

In 2021, as part of its Response to the Royal Commission, the Federal Government agreed to require residential aged care providers to report quarterly on total care staffing minutes by registered nurses, enrolled nurses and personal care workers effective from 1 July 2022.^9^ If successfully implemented, these data may become available in late 2022 or 2023. This requirement signals a significant change in the government's stance, as two previous attempts to legislate for quarterly reporting [the Aged Care Amendment (Staffing Ratio Disclosure) Bill 2018 and the Aged Care Amendment (Staffing Ratio Disclosure) Bill 2019] had failed to gain government support, nor had quarterly reporting been supported by the national Aged Care Workforce Industry Council.^10^

More recently, the Treasurer has tasked the Productivity Commission with examining a specific aspect of the Royal Commission's recommendations relating to ‘employment models in aged care, and the effects that policies and procedures to preference the direct employment of aged care workers would have on the sector’.^11^ The study is focussed on nurses and personal care workers in indirect employment (i.e., agency or subcontractor workers). According to the most recent Department of Health census, this group appears to constitute only 1% of the aged care workforce, although both the Productivity Commission and the Senate Select Committee on Job Security have recently argued that the proportion of indirect employment is likely to be much higher, noting the number of non‐PAYG workers was reportedly substantially higher in 2016 than in 2020, despite widespread reports of an increased need for agency and labour hire staff due to COVID‐19 in 2020.^11^, ^12^

It is tempting to question the value of yet another study of the aged care workforce in the wake of the Royal Commission and the last 17 years of government reports and inquiries on the topic. Given the current crisis in aged care staffing, one might reasonably argue for action rather than investigation. However, there is evidence of significant gaps and anomalies in our national data on the aged care workforce, which means there are gaps in our understanding of what is happening, which in turn affect our capacity to develop solutions in both policy and practice. These gaps and anomalies are the central focus of this article, with a particular emphasis on the terms and conditions of employment, casualisation and insecure employment in residential aged care.

## 2 METHODS

The data presented in this article are a compilation of data from multiple sources. They involve original secondary analysis of data from the following sources: the 2012 and 2016 National Aged Care Workforce Census and Surveys (NACWCS),^13^, ^14^ the 2020 Aged Care Workforce Census,^15^ and the Australian Bureau of Statistics Characteristics of Employment 2020,^16^ in combination with data previously reported by Laß and Wooden from the Household, Income and Labour Dynamics in Australia Survey (HILDA), and the Australian Bureau of Statistics Characteristics of Employment 2016.^17^ An appropriate exemption was obtained from the relevant university ethics committee at the University of Canberra.

## 3 RESULTS

### 3.1 What we know

The most recent data derive from 2020 Aged Care Workforce Census managed by the Department of Health.^15^ According to these data, there were 195,307 nursing and personal care staff in direct care roles in residential aged care, up from the 146,278 reported in 2016 (or 160,394 if the supplementary data on non‐PAYG staff are included).

The four National Aged Care Workforce Census and Survey (NACWCS) reports completed by Flinders University between 2003 and 2016 are routinely quoted as accurate statistical portrayals of the aged care workforce, and the Department of Health 2020 aged care census doubtless, will be as well. There are, however, significant and disturbing gaps in our knowledge deriving from scope, disruptions to the time series as a result of methodological changes, reduced reliability resulting from declining response rates, and the historical weighting system.

The 2020 Census constitutes a break from previous iterations of data collection conducted by Flinders University in regard to the treatment of agency and contract staff. The 2003 to 2012 NACWCS collections focussed primarily on PAYG workers, with additional supplementary information collected in 2016 on non‐PAYG (typically agency) staff. By contrast, the 2020 collection scope was expanded to include agency staff, effectively an improvement in range but which simultaneously reduced our ability to map key workplace trends on something as straightforward as numbers, and the role of residential aged care workers. The publicly available data do not record the change in ways that facilitate reliable comparison.

The 2020 Census also changed the previous approach by dropping the data collection survey of aged care staff that had traditionally been part of the Flinders NACWCS surveys, relying entirely on data provided by facilities. While COVID‐19 undoubtedly brought difficulties for such a collection, the value of knowing what staff report and what their views are is particularly important during confronting times. The response rate in the 2020 ‘census’ was comparatively low at 49%, down from 76% in 2016 and 96% in 2012.^13^, ^14^, ^15^ This substantial reduction in response rate reduces the confidence that can be placed on the reliability of the national estimates put forward on the basis of weighting.

The same weighting method was used across all five collections, taking account of size and geographic location (urban, regional and so on). However, it does not include factors that we know are linked to staffing patterns, such as ownership (public versus for‐profit versus not‐for‐profit), and State or Territory, an issue that was of less relevance at the higher response rates of earlier years, but more likely to affect the accuracy of the findings in 2020. Taken together, these factors mean that while these data are valuable, they should be approached with caution, underlining the importance of the proposed national quarterly reporting on what care is being provided and by whom in residential aged care.

### 3.2 What we do not know

While these basic data on aged care workforce numbers and occupation have limitations, the factors described above impact even more strongly in relation to the terms and conditions of employment for aged care workers.

The 2020 Aged Care Workforce Census reported that 71% of the direct care workforce in residential aged care were permanent part‐time, 6% were permanent full‐time, 19% were casual or contract workers, and 3% were agency staff. The 2016 NACWCS data are similar in relation to the percentage of permanent part‐time workers (71%), but differ in relation to permanent full‐time (11%), casual and contract workers (9%), and agency staff (9%). Earlier trend data showed an increase in the proportion of permanent full‐time and permanent part‐time staff and a decline in the proportion of casual and contract staff from 2012 to 2016, a decline that appears to have been completely reversed in the 2020 Census. Taken together, this reversing pattern raises concerns about the accuracy of the 2020 data, and makes it difficult to place confidence in any conclusions about trends. One clear overarching finding, however, appears to be that the majority of the workforce is employed in permanent roles, and that a modest proportion of persons are employed under casual employment conditions.

When the 2016 NACWCS data are compared to national workforce data, the residential aged care workforce has substantially more permanent part‐time and substantially fewer permanent full‐time workers and roughly half the number of casuals, but this picture is muddied by the unclear role played by the 9% of agency staff. In contrast, the 2020 data are very similar to the 2020 Australian Bureau of Statistics (ABS) data in relation to the proportion of casual staff (19% and 18%), suggesting either an increase in the proportion of casual over this time or an inconsistency in the aged care labour force time series (Table 1).

**TABLE 1:** Employment arrangements of direct care staff (Aged Care Workforce Census and NACWCS) and comparator data from the Australian Bureau of Statistics 2016 and 2020 and HILDA 2016 (headcounts)

	Permanent full‐time %	Permanent part‐time %	Casual or contract %	Agency %	Other (inc. self‐employed) %
Aged Care Workforce Census 2020^a^	6	71	19	3	
NACWCS^a^ 2016	11	71	9	9	1
NACWCS^a^ 2012	9	64	17	10	1
ABS CoE Survey all persons 2020	51	14	18	n.a	17
ABS CoE Survey all persons 2016	48	13	21	n.a.	21
HILDA Survey all persons 2016	45	12	22		20

Source: Data compiled or analysed by the author from the following: Aged Care Workforce Census 2020,^15^ NACWCS 2012 and 2016,^13^, ^14^ Australian Bureau of Statistics Characteristics of Employment 2016,^17^ HILDA 2016,^17^ and Australian Bureau of Statistics Characteristics of Employment (ABS CoE) 2020.^16^

^a^ Residential aged care workforce.

At 19%, the proportion of casual workers reported in the 2020 Census is lower than the national average from the 2016 HILDA data (22%) or the 21% of workers in the ABS employment data for 2016.^17^ It is, however, higher than the 15% reported by community and residential care staff in the 2018 Health Employees Superannuation Trust Association Australia survey.^18^ These comparisons suggest a lack of clarity concerning the proportion of staff in casual employment in the aged care sector, as well as the proportions in indirect employment as raised by the Productivity Commission.^11^ What is even less clear is whether or not the percentages of casual workers and agency workers are an accurate indication of the stability of employment conditions in the aged care workforce. At the very least, these data taken together challenge the all‐too‐frequent assertion that the majority of the aged care workforce are employed on a permanent basis (whether part‐time or full‐time).

## 4 DISCUSSION

While casual employment has often been used as a proxy measure for precarious or insecure employment, permanent part‐time work has generally been regarded as less prone to the concerns of earnings insecurity and working time that characterise casual work.^17^, ^19^, ^20^ In Australia, there is little national evidence on the precarity of permanent part‐time work in residential aged care, although there are indications from the community care sector,^21^ and Charlesworth and Heap^22^ have argued that casualised work practices may emerge within permanent part‐time work. As Campbell and colleagues have argued^23^:

Neglect of minimum‐hour arrangements within permanent work is particularly unfortunate, since such work, despite the ‘permanent’ label, is also precarious and is often associated with the same sort of negative consequences as on‐demand casual work (p. 68).

There are indications that the residential aged care workforce is subject to considerable earnings and working‐time insecurity. According to the staff survey component of the 2016 NACWCS, 30% of the direct care workforce wanted to increase the hours they were working,^14^ a sharp comparison to the 15% reported by the ABS for all employed persons.^16^ The 2016 NACWCS reports that 9% of employees had more than one job,^14^ although this figure may well be higher as these data do not include agency staff. A major industry peak body, Leading Age Services Australia, estimated between 20% and 30% of the aged care workforce employed by their members worked at multiple sites.^24^ Even those with ‘one job’ may work across multiple sites for the same employer, and across both residential and community care services. Staff are known to supplement their income from multiple sources when their pay is insufficient from one location, or from one employer.^21^, ^25^ The pattern is explicitly recognised in the recent COVID‐19‐related measures put in place in Victoria (the Australian state worst affected by cross‐infection and COVID‐19‐related deaths in aged care services) in 2020 to reduce the need for aged care workers to work across multiple sites, while ensuring that no individual worker is adversely affected.^26^ Nonetheless, while the role of financial factors in driving staff to work across multiple sites may have been highlighted, we continue to have very limited evidence on its nature and extent.

Furthermore, there are no data on the proportion of permanent part‐time staff working on zero or minimum hours contracts, where the employer's commitment to hours may vary from 0 to 72 hours. There has been considerable publicity around zero hours contracts in the UK, and the practice has been banned in New Zealand, but in Australia it has been poorly understood.^19^ There is little information about on‐call arrangements whether as part of casual or permanent part‐time work, other than indications that they exist.^27^, ^28^ It may be that the extensive employment of aged care workers under permanent part‐time arrangements masks a form of ‘de facto casualisation’. This makes it difficult to understand what is meant by permanent part‐time work, and, consequently, the precarity of employment faced by aged care workers.

While this issue is an important one for aged care staff, it is also a concern for the residents for whom they care. It is difficult to know, for example, what proportion of staff are likely to be familiar with particular residents or particular wings or nursing homes. These conditions of work matter to staff, and they also matter to residents because the conditions of work influence staff capacity to provide high‐quality care.^29^

These gaps in our understanding of the aged care workforce are problematic and are not addressed by the current national aged care workforce collections. The Federal Government requirement that residential aged care providers report quarterly on total care staffing minutes by registered nurses, enrolled nurses and personal care workers from 1 July 2022 will provide valuable information, but will not improve our understanding of their conditions of employment. The solution may lie in either specific research projects of sufficient scale to accurately document the scale and scope of the issue, or in creative strategies to make use of automatically generated data. This paper has drawn and compared multiple iterations of the national aged care workforce census, together with published HILDA data and the ABS Conditions of Employment data to demonstrate important contradictions and gaps in our knowledge. Having demonstrated these shortcomings, the next step, and one urgently needed for policy purposes, is to gain a more accurate picture.

The Productivity Commission could contribute by innovative analyses of more closely held national data resources. For example, the Australian Bureau of Statistics already uses quarterly payroll data from the Australian Taxation Office to create detailed time series data on payroll jobs and wages. More access to and analysis of the data held in the Australian Taxation Office's Single Touch Payroll (STP) system could prove a valuable source of more accurate information on what is happening in aged care. The STP system was rolled out in 2018 to larger employers, and in 2019 to those with 19 or fewer employees. Phase 2 of the STP rollout commenced in 2022, with more detailed data on employment and taxation conditions, disaggregation of gross income and details of when and why employees leave. It is a mandatory reporting system and covers full‐time, part‐time and casual employees. (Contractors are, however, only captured if included in the employer's payroll software system.) These data may provide reliable evidence on matters such as multiple employers (through linking employee tax file numbers), and on casualisation and variations in hours, all indicators of insecure employment.

Aged care in Australia would benefit from the advice and expertise of the Productivity Commission in identifying and exploring such innovative data sources and in testing their utility. If these options are not viable, then a new research‐based data collection is required. Such activities and recommendations could shed light on optimum data sources and analysis strategies in relation to insecure employment as well as the Productivity Commission's nominated priority area of indirect employment.

## 5 CONCLUSIONS

Australia needs better data on its aged care workforce if the labour shortages confronting the sector are to be better understood and addressed. The recommendations of the Royal Commission on skills training, regulation, remuneration and particularly staffing ratios are all critical—but the conditions of work are also of importance if issues of retention and attraction are to be addressed. Better data on workforce employment conditions are needed to improve understanding of the nature of casual agency and permanent part‐time work in this sector if the looming crisis in aged care workforce is to be averted. While the Productivity Commission has clearly signalled its focus on the small proportion of staff in indirect employment, recommendations and conclusions that elucidate the need for better data on the conditions of employment of a broader subset of the workforce would be beneficial. There are two potential pathways—a special‐purpose high‐quality aged care workforce data collection or innovative use of mandatory national data collections, of which the STP system may be one promising example.

Information of this kind can be used in its turn to develop policies and practices that reduce reliance on casual and agency staff, and consequently, reduce the proportion of staff who are unfamiliar with the care and social needs of residents. High‐quality residential aged care is not only about the number and qualifications of staff, but also about the proportion of permanent staff necessary for the natural human relationships that build with familiarity. Of course, a degree of flexibility in staffing will always be necessary. Nevertheless, holistic person‐centred care requires a high proportion of staff who are familiar with the residents they are caring for, rather than delegation by task to a heavily non‐permanent, itinerant workforce.

## CONFLICTS OF INTEREST

No conflicts of interest declared.

## Data Availability

All data used in this study are publicly accesible on line from the relevant documents and data repositories.
